# Sphingosine 1-phosphate release from platelets during clot formation: close correlation between platelet count and serum sphingosine 1-phosphate concentration

**DOI:** 10.1186/1476-511X-12-20

**Published:** 2013-02-18

**Authors:** Yoshikazu Ono, Makoto Kurano, Ryunosuke Ohkawa, Hiromitsu Yokota, Koji Igarashi, Junken Aoki, Minoru Tozuka, Yutaka Yatomi

**Affiliations:** 1 Department of Clinical Laboratory, The University of Tokyo Hospital, Tokyo, Japan; 2 Department of Clinical Laboratory Medicine, Graduate School of Medicine, The University of Tokyo, 7-3-1 Hongo, Bunkyo-ku, Tokyo, 113-8655, Japan; 3 Analytical Laboratory Chemistry, Graduate School of Health Care Sciences, Tokyo Medical and Dental University, Tokyo, Japan; 4 Bioscience Division, Reagent Development Department, AIA Research Group, TOSOH Corporation, Kanagawa, Japan; 5 Laboratory of Molecular and Cellular Biochemistry, Graduate School of Pharmaceutical Sciences, Tohoku University, Miyagi, Japan

**Keywords:** Sphingosine 1-phosphate, Dihydrosphingosine 1-phosphate, Platelets, Red blood cells, Autotaxin

## Abstract

**Background:**

Sphingosine 1-phosphate (Sph-1-P), abundantly stored in platelets and released extracellularly upon activation, plays important roles as an extracellular mediator by interacting with specific cell surface receptors, especially in the area of vascular biology and immunology/hematology. Although the plasma Sph-1-P level is reportedly determined by red blood cells (RBCs), but not platelets, this may not be true in cases where the platelets have been substantially activated.

**Methods and results:**

We measured the Sph-1-P and dihydrosphingosine 1-phosphate (DHSph-1-P) levels in serum samples (in which the platelets had been fully activated) from subjects with (n = 21) and without (n = 33) hematological disorders. We found that patients with essential thrombocythemia exhibited higher serum Sph-1-P and DHSph-1-P concentrations. The serum Sph-1-P concentration was closely correlated with the platelet count but was very weakly correlated with the RBC count. Similar results were obtained for DHSph-1-P. The serum Sph-1-P and DHSph-1-P levels were inversely correlated with the level of autotaxin (ATX), a lysophosphatidic acid-producing enzyme. A multiple regression analysis also revealed that the platelet count had the greatest explanatory impact on the serum Sph-1-P level.

**Conclusions:**

Our present results showed close correlations between both the serum Sph-1-P and DHSph-1-P levels and the platelet count (but not the RBC count); these results suggest that high concentrations of these sphingoid base phosphates may be released from platelets and may mediate cross talk between platelet activation and the formation of atherosclerotic lesions.

## Introduction

Sphingosine 1-phosphate (Sph-1-P) has been shown to be an important lipid mediator in the fields of vascular biology and immunology/hematology [1]. Although Sph-1-P has been demonstrated to act as an intracellular mediator, most of the physiological functions of Sph-1-P are exerted through S1P receptors located on the plasma membrane [2,3]. Therefore, elucidating the mechanism that regulates plasma Sph-1-P levels is an important task. Regarding the source of Sph-1-P in plasma, a previous report proposed that the plasma circulating Sph-1-P level might be determined by platelets, since Sph-1-P is abundantly accumulated in platelets [4]. However, from experiments using genetically modified mice models [5] and analyses in human subjects [6], the plasma Sph-1-P level is now thought to be determined not by platelets, but mainly by erythrocytes. This theory seems plausible because the circulating volume of erythrocytes is much greater than that of platelets.

Although erythrocytes determine the plasma Sph-1-P levels during the steady state in healthy subjects, however, platelets may also play an important role during pathological states in which the platelets are activated, such as in thrombosis. Regarding this possibility, the clinical significance of Sph-1-P in atherosclerotic diseases differs depending on the type of samples in which the Sph-1-P level is measured: plasma HDL-linked Sph-1-P, albumin-linked Sph-1-P, or serum Sph-1-P. Clinical studies investigating Sph-1-P levels in atherosclerotic diseases have reported that although HDL linked Sph-1-P is negatively related with atherosclerosis [7,8], Sph-1-P in non-HDL fractions and serum Sph-1-P are positively correlated with atherosclerosis [7,9]. This discrepancy might be explained by the different origin of Sph-1-P in these samples; contrary to plasma samples in which the Sph-1-P levels are mainly determined by erythrocyte-derived Sph-1-P, platelets can have a large impact on the Sph-1-P concentration in serum samples in which the platelets are fully activated. If true and considering that Sph-1-P released from platelets might be preferably carried on albumin (non-HDL fraction) [10,11], these results suggest that platelet-derived Sph-1-P might exert proatherosclerotic properties. To our knowledge, the factors that determine the serum Sph-1-P levels have not yet been elucidated. In the present study examining serum samples from subjects with and without hematological disorders, we investigated the factors that determine the serum Sph-1-P levels. We also examined dihydrosphingosine 1-phosphate (DH-Sph-1-P), which is considered to work as an agonist against Sph-1-P receptors [12].

## Results

### Serum Sph-1-P and DHSph-1-P levels were increased in patients with essential thrombocythemia (ET)

**Figure 1:**
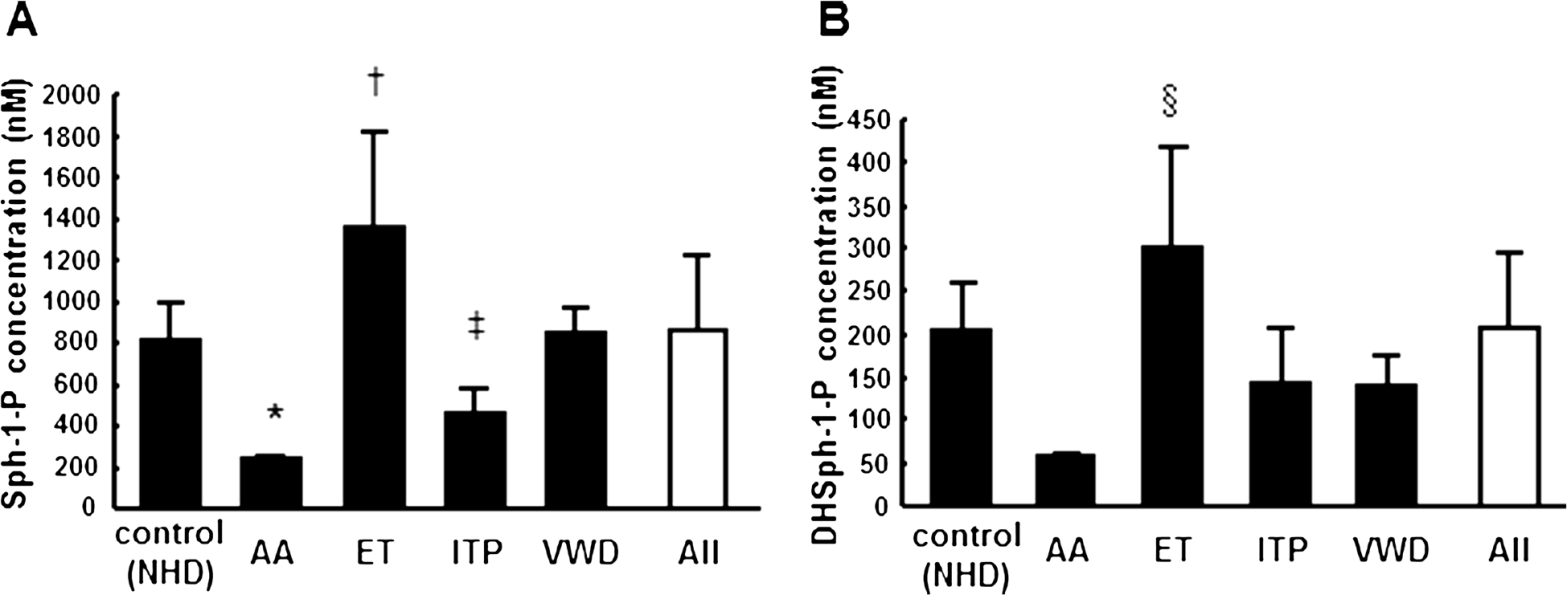
**Serum Sph-1-P and DHSph-1-P levels in non-hematological disorder subjects and subjects with hematologic diseases.** Serum samples were collected from the patients described in Table 1 and the serum Sph-1-P **(A)** and DHSph-1-P levels **(B)** were measured. *P<0.05 vs. the NHD (non-hematological disorder) group and *P*<0.01 vs. ET (essential thrombocythemia). †*P*<0.01 vs. AA (aplastic anemia) and NHD and *P*<0.05 vs. VWD (von Willebrand diseases), ‡*P*<0.05 vs. NHD and *P*<0.01 vs. ET, §*P*<0.01 vs. NHD, AA, and ITP (idiopathic thrombocytopenia purpura) and *P*<0.05 vs. VWD.

To elucidate which factors determine the serum Sph-1-P and DHSph-1-P levels, we first measured the serum Sph-1-P and DHSph-1-P levels in subjects with hematological disorders and with non-hematological disorders (NHD) (Figure 1). The basic characteristics of the subjects are shown in Table 1. As predicted by the fact that erythrocytes determine the plasma Sph-1-P levels to a large degree [6], the serum Sph-1-P levels were lower in the aplastic anemia (AA) group, which had a significantly lower RBC count, compared with the other groups. Importantly, the ET group had a significantly higher Sph-1-P and DHSph-1-P level, although the RBC count was similar to those of the other groups except for the AA group. The platelet count for the ET group was much higher than those in the other groups (Table 1). Therefore, these results suggested that platelet count might also determine the serum Sph-1-P and DHSph-1-P levels.

**Table 1:** Basic characteristics of the subjects

	**RBC (X10**^**4**^**/μL)**	**Platelets (X10**^**4**^**/μL)**	**alb (mg/dL)**	**HDL-C (mg/dL)**	**LDL-C (mg/dL)**	**TG (mg/dL)**	**ATX (ng/mL)**
AA	215.0 ± 9.9*	2.8 ± 0.5	4.4 ± 0.1	54.8 ± 14.6	103.6 ± 35.4	178.5 ± 200.1	1.49 ± 0.39
ET	467.9 ± 101.2	84.8 ± 35.4†	4.2 ± 0.2	56.8 ± 14.4	104.5 ± 40.4	144.7 ± 66.5	0.96 ± 0.13
ITP	472.2 ± 40.1	3.1 ± 1.1	4.1 ± 0.4	57.9 ± 15.7	100.9 ± 25.9	164.2 ± 127.3	0.96 ± 0.13
VWD	423.0 ± 89.9	27.5 ± 6.3	4.4 ± 0.2	28.9 ± 0.4	73.9 ± 59.9	127.7 ± 29.6	0.95 ± 0.12
NHD	436.4 ± 71.8	22.1 ± 8.2	4.1 ± 0.5	40.3 ± 19.0	127.4 ± 53.5	135.7 ± 71.7	0.90 ± 0.07
All	437.3 ± 86.6	31.2 ± 31.1	4.1 ± 0.4	45.1 ± 18.9	116.0 ± 49.6	140.3 ± 78.7	0.95 ± 0.05

Serum samples were collected from subjects with NHD (non-hematological disorders, n=33) and patients with AA (aplastic anemia, n=2), ET (essential thrombocythemia, n=10), ITP (idiopathic thrombocytopenia purpura, n=6), and VWD (von Willebrand diseases, n=3). ATX represents autotaxin. Data were shown as the mean ± SD.

* *P*<0.01 vs. ET, ITP, NHD, and *P*<0.05 vs. VWD; †*P*<0.01 vs. the other groups.

### Serum Sph-1-P and DHSph-1-P concentrations were more positively related with the platelet count than with the RBC count

**Figure 2:**
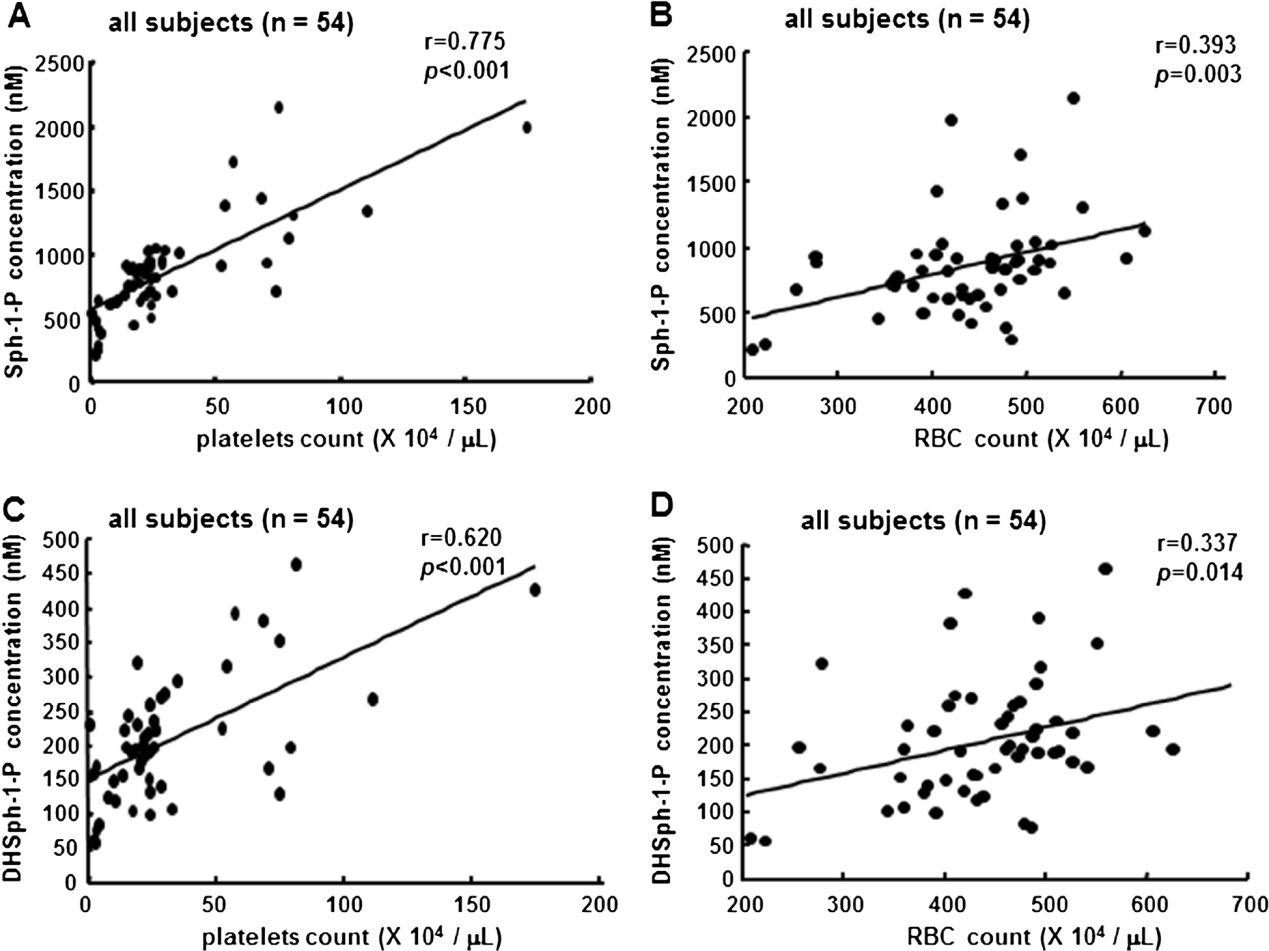
**Correlations between serum Sph-1-P level or serum DHSph-1-P level and platelet count or RBC count in all subjects.** The correlations between the serum Sph-1-P level and the platelet count **(A)** and the RBC count **(B)** and between the serum DHSph-1-P level and the platelet count **(C)** and the RBC count **(D)** in all subjects (n = 54) are shown.

Next, we compared the serum Sph-1-P and DHSph-1-P levels with the RBC count or the platelet count. As shown in Figure 2B, the serum concentration of Sph-1-P was significantly correlated with the RBC count (r = 0.393, *P* = 0.003). However, unlike the plasma Sph-1-P level, the serum Sph-1-P level was more significantly correlated with the platelet count (r = 0.775, *P*<0.001, Figure 2A) than with the RBC count. Similar results were observed for the serum DHSph-1-P level (Figure 2C and D). These results suggested that the serum Sph-1-P and the DHSph-1-P levels might derive largely from activated platelets in prepared serum samples.

**Figure 3:**
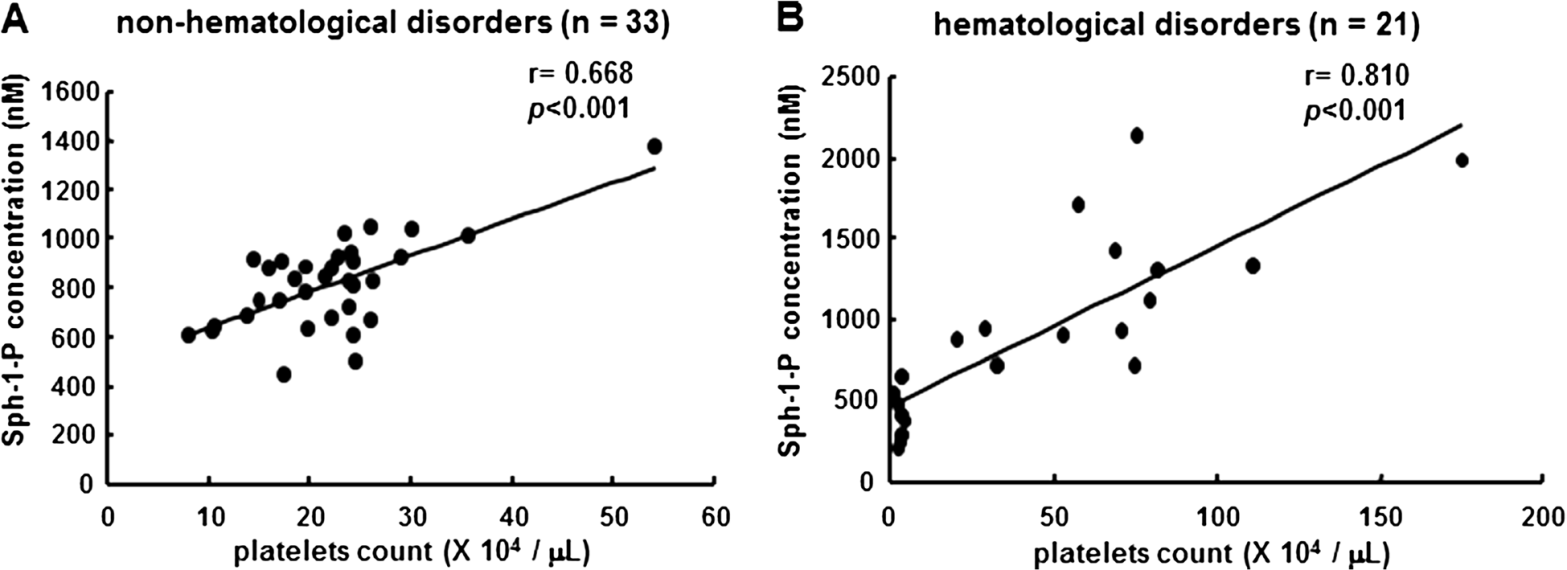
**Correlations between serum Sph-1-P level and platelet count in subjects and in subjects with NHD and with hematological disorders.** The correlations between the serum Sph-1-P level and the platelet count in NHD subjects (n = 33) **(A)**, and in subjects with hematological disorders (n= 21) **(B)** are shown.

We also evaluated the correlation between the serum Sph-1-P level and the platelet count in NHD subjects or subjects with hematological disorders separately. In samples from NHD subjects alone, a close correlation between the Sph-1-P or DHSph-1-P level and the platelet count was observed (r = 0.668, *P*<0.001, Figure 3A; r = 0.587, *P*<0.001, respectively). Also in samples from subjects with hematological disorders, a significant close correlation between the Sph-1-P level and the platelet count was observed (r = 0.810, *P*<0.001, Figure 3B). These results suggest that the serum Sph-1-P and DHSph-1-P levels are mainly determined by the platelet count irrespective of the presence of hematological disorders.

### Serum Sph-1-P and DHSph-1-P concentrations were inversely related with the serum ATX level

We further investigated possible determinants of the Sph-1-P and DHSph-1-P concentrations. First, we investigated the correlation of Sph-1-P or DHSph-1-P with the HDL cholesterol and albumin levels, since Sph-1-P is mostly bound to HDL or albumin in plasma [13,14]. However, no significant correlations were observed with HDL cholesterol and albumin levels (data not shown).

**Figure 4:**
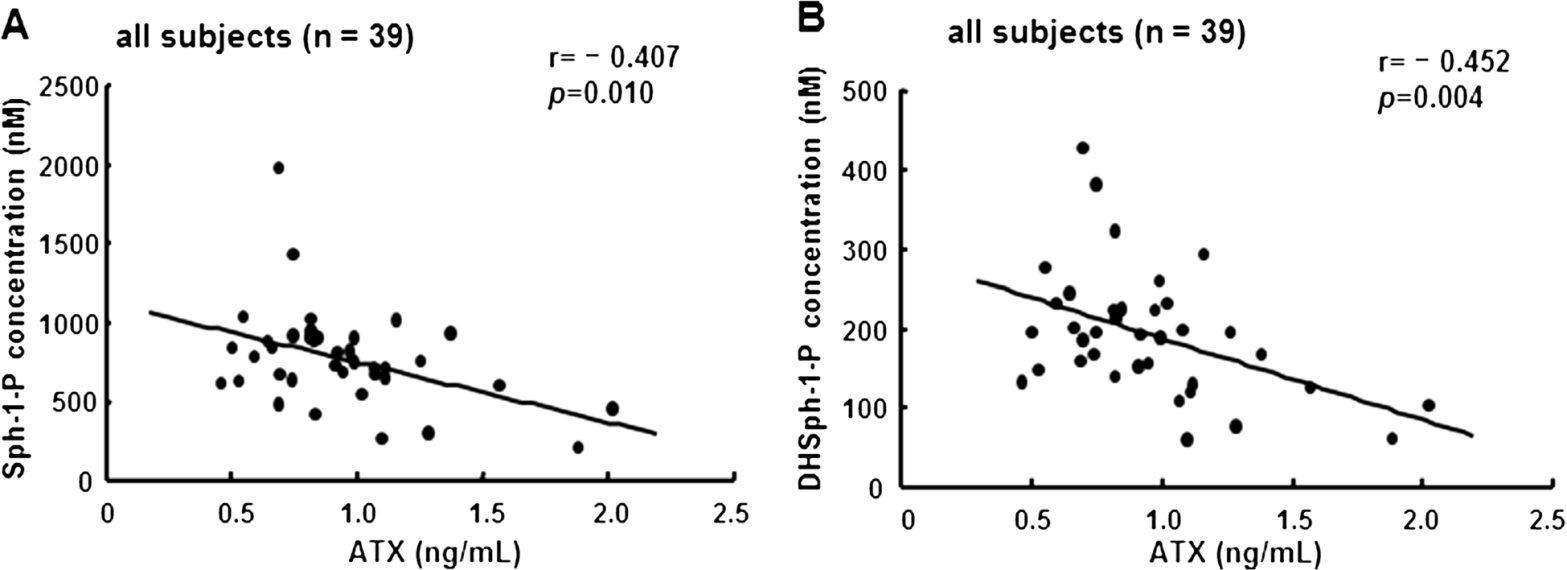
**Correlations between the serum Sph-1-P level or the DHSph-1-P level and the ATX level.** The correlations between the serum Sph-1-P level **(A)** or the DHSph-1-P **(B)** level and the ATX level in all subjects (n = 39) are shown.

We next evaluated the correlation between Sph-1-P or DHSph-1-P and the autotaxin (ATX) level. ATX is a key enzyme in the production of lysophosphatidic acid (LPA) from lysophosphatidylcholine (LPC) [15,16]. Along with LPA synthesis, Sph-1-P and DHSph-1-P can theoretically be formed with ATX from sphingosylphosphorylcholine (SPC) [17]. However, contrary to our expectations, the serum ATX level was significantly and inversely related to the serum Sph-1-P and DHSph-1-P concentrations (r = -0.407, *P* = 0.010 and r = -0.452, *P*<0.001, respectively) (Figure 4A and B).

### Multiple regression analysis revealed that the platelet count was the most important explanatory variable determining the serum Sph-1-P and DHSph-1-P concentrations

Finally, we performed a multiple regression analysis utilizing the serum Sph-1-P or DHSph-1-P level as the objective variable, as mentioned in the Materials and methods section. As predicted from the correlation studies, the platelet count was the most important explanatory variable for the Sph-1-P concentration, followed by the ATX level and RBC count in this order (Table 2). A similar result was obtained for DHSph-1-P, except for the RBC count (Table 3).

**Table 2:** Multiple regression analysis for serum Sph-1-P level in all the subjects

	**b 95% CI**	**Standardized β**	***P *value**
Platelets	8.17 (6.44 - 9.91)	0.791	<0.001
ATX	−196.40 (−361.46 - −31.35)	−0.219	0.021
RBC	0.78 (0.10 - 1.47)	0.208	0.027
HDL-C			0.196
TG			0.312
LDL-C			0.721
Albumin			0.857

**Table 3:** Multiple regression analysis for serum DHSph-1-P level in all the subjects

	**b 95% CI**	**Standardized β**	***P *value**
Platelets	1.41 (0.73 - 2.09)	0.532	<0.001
ATX	−86.20 (−145.04 - −27.37)	−0.378	0.021
RBC			0.253
HDL-C			0.703
TG			0.223
LDL-C			0.604
Albumin			0.604

## Discussion

Sphingosine 1-phosphate (Sph-1-P) plays important roles as an extracellular mediator through its interactions with the G protein-coupled receptor S1P1-5, which is expressed on the cell surface; these actions are especially pertinent in the fields of vascular biology, immunology, and hematology. Although erythrocytes are the major determinant of the plasma Sph-1-P level [5,6], the major determinant of the serum Sph-1-P concentration has not yet been elucidated. In the present study, we attempted to elucidate the source of serum Sph-1-P. We also investigated the determinants of the DHSph-1-P concentration, which are thought to act similarly as a ligand for Sph-1-P receptors as Sph-1-P [12].

In this study, we demonstrated that the serum Sph-1-P level was closely related to the platelet count (Figure 2). This finding seems to be reasonable because when the results were adjusted according to the phospholipid level, the platelets contained a higher concentration of Sph-1-P than the erythrocytes [4] and the platelets were fully activated in the serum samples. Multiple regression studies also revealed that the platelet count was the most explanatory variable for Sph-1-P (Table 2).

To our knowledge, this report is the first to demonstrate that the serum Sph-1-P level is determined by the platelet count. This result provides important clues related to the physiological properties of Sph-1-P in atherosclerotic diseases. In the physiological state, cells involved in atherogenesis, such as endothelial cells, macrophages, and smooth muscle cells, might be exposed to Sph-1-P concentrations corresponding to the plasma Sph-1-P level (300-500 nM). However, as reported in this study, the Sph-1-P concentration in serum samples in which the platelets are fully activated was much higher (around 800 to 1,000 nM) than the plasma Sph-1-P level. Therefore, cells are likely exposed to higher concentrations of Sph-1-P at the exact site where thrombosis is occurring. This difference in the Sph-1-P concentrations between plasma and the exact site of thrombosis may explain the apparently contradictory clinical influences of serum Sph-1-P and plasma Sph-1-P on atherosclerosis [7-9]. According to the results from in vitro experiments, Sph-1-P exerts anti-atherosclerotic properties such as the induction of NO production [18], the suppression of TNF-?-induced VCAM-1 induction [19], and the inhibition of the adhesion of monocytes on the endothelium through the rearrangement of endothelial integrins [20] at concentrations up to several hundred nM; meanwhile, Sph-1-P can induce adhesion molecules by activating nuclear factor κ-B [19,21,22] and activate platelets [23,24] at concentrations higher than several μM. Therefore, Sph-1-P can create a vicious cycle formed by local thrombosis in atherosclerotic lesions; platelets are activated in the atherosclerotic lesions and Sph-1-P is released from the platelets, and then the high concentration of Sph-1-P accelerates atherosclerosis and further activates platelets. Further clinical and basic studies are needed to elucidate the association between serum Sph-1-P and atherosclerotic diseases as well as the possible existence of other unknown factors determining the release of Sph-1-P from platelets.

Concerning this issue, interesting relationships between the serum Sph-1-P and ATX levels were observed in the present study. ATX is a key enzyme in the production of LPA; LPA is hydrolyzed from LPC by ATX/lysophospholipase D (LysoPLD) [15,16]. Along with these functions, ATX is known to hydrolyze SPC to produce Sph-1-P [17], although the contribution of this pathway to Sph-1-P might be much smaller than the well-known pathway in which sphingosine is converted into Sph-1-P, considering the concentration of SPC in vivo. Therefore, the observation of an inverse correlation between Sph-1-P and ATX seems contradictory. One possible mechanism is that substrates produced by ATX, such as LPA, might be involved in the formation of Sph-1-P. Further studies are needed to examine this possibility.

In this study, we measured the serum DHSph-1-P level as well as the serum Sph-1-P level. DHSph-1-P was shown to behave as an Sph-1-P receptor agonist similar to Sph-1-P [12]; however, little information is available regarding the modulation of the in vivo DHSph-1-P level. As shown in Figures 1, 2 and 4, and Table 3, we demonstrated that the serum DHSph-1-P level was determined in a manner similar to the Sph-1-P level.

In summary, this study demonstrated that the serum Sph-1-P and DHSph-1-P levels were determined mainly by the platelet count and suggested that the high concentrations of Sph-1-P released from platelets might mediate cross talk between platelet activation and the formation of atherosclerotic lesions.

## Materials & methods

### Serum sample preparation

Non-hematological disorder subjects [NHD] (n = 33) and patients with aplastic anemia [AA] (n = 2), essential thrombocythemia [ET] (diagnosed according to the WHO classification criteria [25]) (n = 10), idiopathic thrombocytopenia purpura [ITP] (n = 6), and von Willebrand diseases [VWD] (n = 3) were enrolled.

The serum samples used in this study were residual samples of those obtained after the completion of routine laboratory analyses. The study was approved by the Institutional Research Ethics Committee of the Faculty of Medicine, the University of Tokyo.

### Measurement of Sph-1-P and DHSph-1-P

Sph-1-P and DHSph-1-P in serum samples were extracted using a two-step extraction method and were measured using high-performance liquid chromatography (HPLC) using C17-Sph-1-P as an internal standard, as previously described [26].

### Measurement of serum autotaxin levels

The autotaxin (ATX) antigen levels in the serum were determined using a two-site immunoenzymetric assay with the ATX assay reagent and the TOSOH AIA system (TOSOH, Tokyo, Japan) [27].

### Statistical analysis

All the data were statistically analyzed using SPSS (Chicago, IL). The results were expressed as the mean ± SD. Values obtained from more than three groups were compared using a one-way analysis of variance (ANOVA) followed by a post-hoc test. Correlations were evaluated using the Pearson correlation. The independent effects of the RBC count, platelet count, albumin, ATX, HDL-C, LDL-C, and TG levels on the serum Sph-1-P or DHSph-1-P levels were evaluated using stepwise multiple regression analyses. P-values less than 0.05 were deemed statistically significant for all the analyses.

## Abbreviations

AA: Aplastic anemia; ATX: Autotaxin; DHSph-1-P: Dihydrosphingosine 1-phosphate; ET: Essential thrombocythemia; HPLC: High-performance liquid chromatography; ITP: Idiopathic thrombocytopenia purpura; LPA: Lysophosphatidic acid; LPC: Lysophosphatidylcholine; LysoPLD: Lysophospholipase D; NHD: Non-hematological disorder; RBC: Red blood cell; Sph-1-P: Sphingosine 1-phosphate; SPC: Sphingosylphosphorylcholine; VWD: Von Willebrand disease.

## Competing interests

The authors declared that they have no competing interests.

## Authors’ contribution

YO and MK participated in study design, carried out experiments and data analysis, and drafted the initial manuscript. RO participated in several experiments. KI participated in measurement of serum autotaxin levels. JA, HY, and MT were involved in study design and drafting manuscript. YY conceived of the study, coordinated the study design and helped to draft the manuscript. All authors read and approved the final manuscript.
